# Shufeng Jiedu capsules for treating acute exacerbations of chronic obstructive pulmonary disease: a systematic review and meta-analysis

**DOI:** 10.1186/s12906-020-02924-5

**Published:** 2020-05-24

**Authors:** Ru-yu Xia, Xiao-yang Hu, Yu-tong Fei, Merlin Willcox, Ling-zi Wen, Ming-kun Yu, Li-shan Zhang, Meng-yuan Dai, Guang-he Fei, Mike Thomas, Nick Francis, Tom Wilkinson, Michael Moore, Jian-ping Liu

**Affiliations:** 1 Centre for Evidence-Based Chinese Medicine, Beijing University of Chinese Medicine, No. 11 North Sanhuan East Road, Chaoyang District, Beijing, 100029 China; 2 School of Primary Care, Population Sciences and Medical Education, University of Southampton, Aldermoor Health Centre, Aldermoor Close, Southampton, SO16 5ST UK; 3 Respiratory Department, Dongzhimen Hospital Affiliated to Beijing University of Chinese Medicine, No.5 Hai Yun Cang, Dongcheng District, Beijing, 100700 China; 4 Department of Respiratory and Critical Care Medicine, First Affiliated Hospital of Anhui Medical University, No.210 Jixi Road, shushan District, Hefei, 230022 Anhui Province China; 5 Clinical and Experimental Sciences, Faculty of Medicine, University of Southampton, Southampton General Hospital, Tremona Road, Southampton, SO16 6YD UK; 6 Institute of Integrated Traditional Chinese Medicine and Western Medicine, Guangzhou Medical University, Guangzhou, 510120 China

**Keywords:** COPD, Exacerbation, Shufeng Jiedu, Systematic review, Meta-analysis, Chinese herbal medicine, Randomised controlled trial

## Abstract

**Background:**

Chinese herbal medicine is widely used in combination with usual care for acute exacerbations of chronic obstructive pulmonary disease (AECOPD) in China. Chinese patent medicine Shufeng Jiedu (SFJD) capsules is widely used for respiratory infectious diseases. This review aims to evaluate effectiveness and safety of SFJD for AECOPD.

**Methods:**

A systematic review of randomised controlled trials (RCTs) in patients with AECOPD, who received SFJD as a single intervention or as add-on treatment to usual care. PubMed, the Cochrane Library, EMBASE, Scopus, Web of Science and four Chinese databases were searched from inception to April 2019. Two authors screened trials, extracted data, and assessed risk of bias, independently. Meta-analysis was performed using RevMan 5.3 software. We performed subgroup analyses and sensitivity analyses according to the predefined protocol. Quality of evidence was assessed using GRADE.

**Results:**

Thirteen RCTs (1036 patients, with 936 inpatients) were included, all compared SFJD in combination with usual care (including antibiotics) to usual care alone. The mean age of participants ranged from 52 to 67 years, with approximately 60% male. Due to lack of blinding and other factors, all trials were of high risk of bias. SFJD was associated with a significant reduction in treatment failure, from 20.1 to 8.3% (11 trials; 815 patients; relative risk 0.43, 95% confidence interval [CI] 0.30 to 0.62), and duration of hospital stay (2 trials; 79 patients; mean difference − 4.32 days, 95% CI − 5.89 to − 2.75 days). No significant difference in adverse events was found between SFJD and control groups.

**Conclusion:**

Low certainty evidence suggests SFJD may bring additional benefit in reducing treatment failure, shorten hospital stay, and improving symptoms. Further large, high quality RCTs are needed to confirm its benefit and safety.

**Trial registration:**

PROSPERO CRD42019133682.

## Background

Chronic obstructive pulmonary disease (COPD) is one of most common causes of impaired health [1]. In China, around 99.9 million people, 8.6% of the Chinese population aged 20 years or older, live with COPD [2]. In the UK there is an estimation of 3 million COPD patients, among whom 1.2 million have been diagnosed, costing the National Health Service over 800 million pounds per year [3, 4]. Acute exacerbations of chronic obstructive pulmonary disease (AECOPD) are defined as an acute worsening of respiratory symptoms that require additional therapy [5–9]. Patients with COPD on average experience 0.5–3.5 acute exacerbations per year [10]. Acute exacerbations lead to a decline in lung function and quality of life, increased need for hospitalisation, and are associated with increased risk of death [11, 12], therefore account for the greatest proportion of the total COPD burden on healthcare system [13, 14].

AECOPD can be triggered by several factors, with the most common causes being respiratory infections caused by bacteria or viruses (which may coexist) and non-infectious environmental factors such as pollution or allergens. Severe exacerbations may require hospitalisation or visits to the emergency department and may be associated with acute respiratory failure. Bronchodilators are commonly prescribed in combination with systemic corticosteroids, antibiotics and other respiratory support in the treatment of AECOPD. These therapies are supported by evidence from randomised controlled trials and are recommended by guidelines [9, 15]. However, these interventions are known to cause a variety of adverse effects, including, dry mouth, tremor, hyperglycaemia, diarrhoea, and antibiotic resistance [16–18].

Shufeng Jiedu (SFJD) capsule is an oral patent Chinese herbal medicine widely used in China for the treatment of respiratory infections (a list of all ingredients of Shufeng Jiedu is available in Additional file 1: Table 1). Published systematic reviews [19–26] have evaluated the effects of many traditional Chinese herbal medicine decoctions and injections on clinical outcomes including symptoms, pulmonary function and quality of life in patients with AECOPD. However, there is no published systematic review evaluating the effectiveness and safety of SFJD as a treatment for AECOPD. In light of the recently published randomised controlled trials (RCTs) on SFJD, we aimed to evaluate the effectiveness and safety of SFJD for AECOPD by conducting a systematic review and meta-analysis where appropriate.

## Methods

This systematic review is reported in accordance with the PRISMA statement [27] and has been prospectively registered on PROSPERO [28].

### Eligibility criteria

We included RCTs of SFJD as a single intervention or in combination with usual treatment (e.g. bronchodilators and antibiotics), compared to usual treatment alone, placebo, waiting list or no treatment. Participants were patients with a clinical diagnosis of AECOPD. The primary outcome was treatment failure as observed at the end of treatment. Treatment failure was defined as no resolution or deterioration of symptoms after trial medication of any duration, or death (when explicitly stated, due to exacerbation) or additional course of antibiotics or another medication for the treatment of AECOPD [17].

Secondary outcomes included all-cause mortality, duration of hospital stay (for inpatients), admission to an intensive care unit (ICU), re-exacerbations within two to 6 weeks of index exacerbation (inpatient or outpatient treatment, rates or time to event), time to resolution of clinical symptoms (e.g. dyspnoea, cough, wheezing, sputum, or fever), arterial blood gas measurements at the end of treatment (partial pressure of oxygen in arterial blood (PaO2) and partial pressure of carbon dioxide in arterial blood (PaCO2)), lung function - forced expiratory volume in 1 s (FEV1) / forced vital capacity (FVC) ratio, markers of infection (white blood cell count, proportion of neutrophils, C-reactive protein, procalcitonin), antibiotic usage (change of modes of administration of antibiotics, change of antibiotic, duration of antibiotic treatment or number of patients who used antibiotics), health related quality of life and adverse events (unintended symptoms or signs, e.g. abnormal liver/ kidney function or ECG, or disease).

### Information sources

We searched English databases PubMed, the Cochrane Library, EMBASE, Scopus, Web of Science, and Chinese databases China National Knowledge Infrastructure (CNKI), Chinese Scientific Journal Database (VIP), SinoMed and Wanfang from their inception to April 2019. The PubMed search used the following words in Title/Abstract field: “Shufengjiedu” or “Shu Feng Jie Du” or “Shu-Feng-Jie-Du” or “Shufeng Jiedu” or “Shufeng-Jiedu”. We conducted searches of ClinicalTrials.gov (www.ClinicalTrials.gov) and Chinese Clinical Trial Registry (http://www.chictr.org.cn/index.aspx). We also searched reference lists of included studies and existing systematic reviews.

### Literature screening, data extraction and quality assessment

After removing duplicates, paired trained reviewers independently screened titles and abstracts of all potential studies identified from searches. When there were uncertainties, insufficient information, or in cases of disagreement, we obtained full texts of the articles, then determined eligibility by screening the full texts. Reasons for excluding articles at full text screening stage were recorded.

After identifying eligible studies, paired reviewers independently extracted trial characteristics on sample size, setting and source of funding, characteristics of the trial population on age and sex, information about the illness including diagnostic criteria and illness duration, trial inclusion and exclusion criteria, details of interventions in all trial arms, outcome measures, and risk of bias domains from included trials using standardised pilot tested forms with detailed instructions.

Quality of the eligible studies was assessed by two reviewers using a modified Cochrane Risk of Bias Tool with four response options: “probably no”, “no”, “probably yes”, and “yes” [29, 30]. We also used the five GRADE (Grading of Recommendations Assessment, Development and Evaluation) considerations (risk of bias, directness, precision, consistency, and the possibility of publication bias) to assess methodological confidence of a body of evidence for prespecified outcomes [31]. For all phases of the review, we dealt with discrepancies through discussion or adjudication by a third reviewer when necessary. Chance-corrected Kappa [32] was calculated as a measure of agreement among reviewers’ judgements.

### Data synthesis and analysis

We conducted analyses using risk ratio (RR) with 95% confidence intervals (CI) for dichotomous data; mean difference (MD) or standard mean difference (SMD) with 95% CI for continuous data. We pooled data quantitatively through Review Manager (version 5.3) when the trials had admissible clinical homogeneity and statistical heterogeneity as measured by Cochrane *χ*^*2*^ test and *I*^*2*^ statistic, or where heterogeneity could be explained by predefined subgroup analysis [29, 33]. Otherwise only qualitative description of the data was presented. A fixed-effects model was considered when *I*^*2*^ was < 30%, otherwise, a random-effects model was utilised.

To explain heterogeneity, we conducted subgroup analyses predefined via AECOPD severity (outpatients, inpatients and patients admitted to the ICU), treatment duration (≤7 days or > 7 days), mode of administration of antibiotics, and complications. We conducted sensitivity analyses to challenge the robustness of the results when there were clinically meaningful differences in primary outcomes considering: multi-centre versus single centre, clear versus unclear randomisation concealment, placebo used versus not used, reported loss-to-follow-up versus not reported, assumed worst plausible case results for patients in intervention groups with missing data [34].

We generated a funnel plot through Review Manager (version 5.3), and performed Begg’s test and Egger’s test through R (version 3.6.1) when ten or more studies were presented in a meta-analysis to examine publication bias.

## Results

**Fig. 1:**
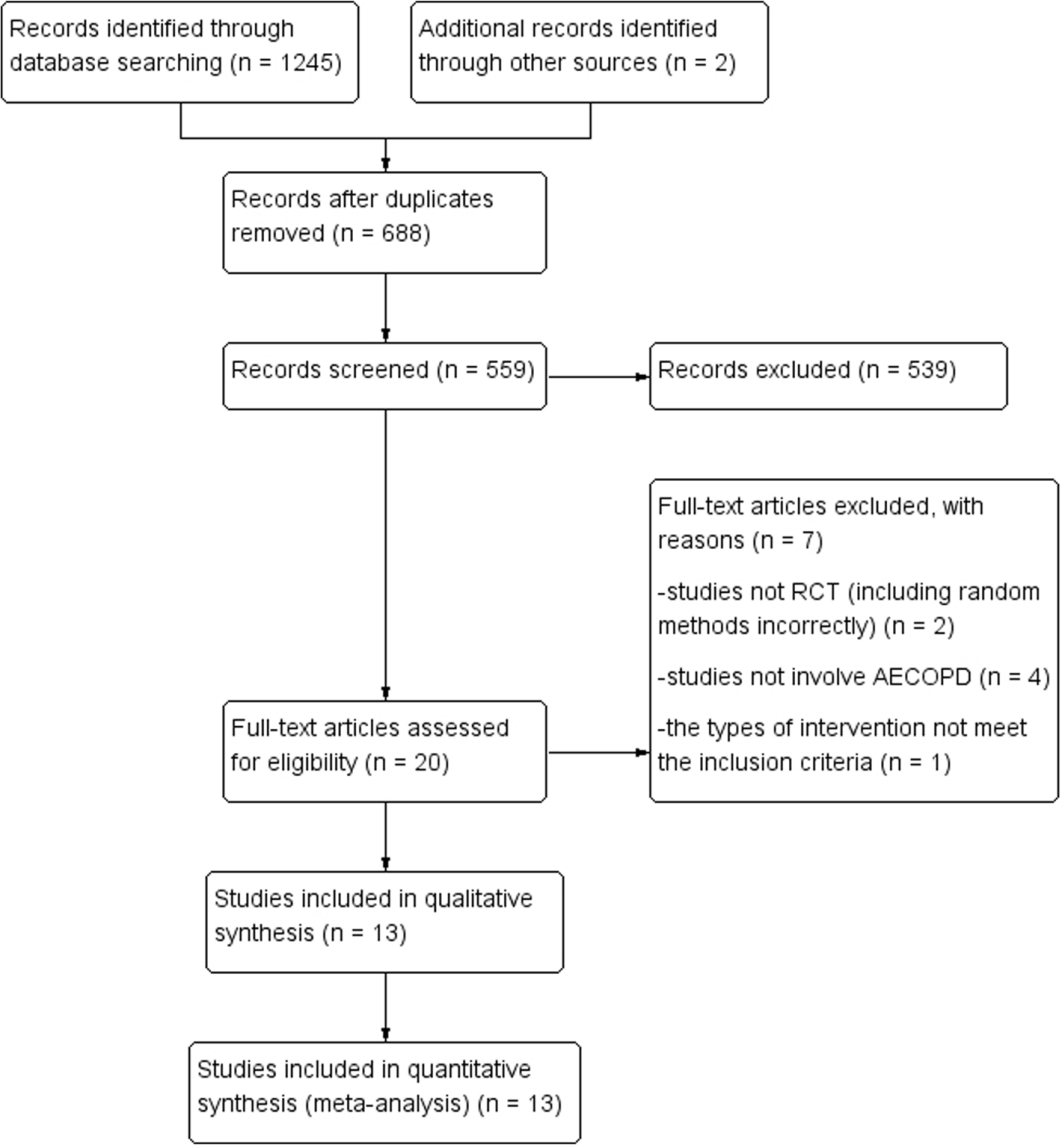
Flow diagram. RCT: randomised controlled trial; AECOPD: acute exacerbations of chronic obstructive pulmonary disease

The literature search identified 688 unique citations, among which 20 were judged potentially eligible at title and abstract screening. Further screening of full text identified 13 RCTs involving 1036 patients, of which 936 were hospitalised patients (Fig. 1). Chance-corrected *Kappa* for agreements is 0.95 for title and abstract screening, 0.77 for full text screening, and 0.91 for assessment of risk of bias of included studies.

### Trial characteristics

All studies were single centre trials and conducted in China, and none reported sources of funding or conflicts of interest. Sample size ranged from 40 to 130, more than half of the participants were male in each trial, and the mean age ranged from 52 to 67 years (Table 1) [35–47]. Patients in intervention groups all received SFJD along with antibiotics and symptomatic treatments (such as bronchodilators and supplemental oxygen), while the comparison groups all received antibiotics and symptomatic treatments without SFJD. The duration of treatment ranged from 6 [47] to 14 days [41, 46]. In all included trials, the dose of SFJD was 4 capsules per time, 3 times daily.

**Table 1:** Characteristics of included trials

Study ID	Outpatient /inpatient	Exacerbation occur within n hours of study enrolment	Funding	Sample size	Average age (year)	Male (%)	Treatment		Use of placebo	Treatment duration (days)	Outcome measurements ^b^
							Add-on treatment in experimental group ^a^	Usual treatment for both groups			
Bian 2016 [35]	Outpatient	< 24 h	NR	50/50	62	73	SFJD	Levofloxacin + ST	No	7	6;7;8
Hu 2018 [36]	Inpatient	NR	NR	60/60	63.1/63.8	53.3/52.9	SFJD	Antibiotic + ST	No	10	1;3;5;6
Huang 2015 [37]	Inpatient	< 48 h	NR	45/45	66/67	66.7/77.1	SFJD	Cefoperazone and sulbactam + ST	No	10	1;5;7;9
Li 2017a [38]	Inpatient	NR	NR	20/20	67.2/66.1	80/80	SFJD	Antibiotic + ST	No	7	1;2;9
Li 2017b [39]	Inpatient	< 48 h	NR	50/50	52.3/54.7	74/70	SFJD	Ceftriaxone (iv.gtt, 2 g/d) + ST	No	10	1;4;7;9
Tian 2018 [40]	Inpatient	NR	NR	43/43	61.3/60.5	65.1/60.5	SFJD	β-lactams + ST	No	7	1;9
Wang 2016 [41]	Inpatient	< 48 h	NR	41/39	63.5/62.5	56.1/48.7	SFJD	Antibiotic + ST	No	14	1
Wang 2018 [42]	Inpatient	≤48 h	NR	35/35	54.2/52.3	65.7/71.4	SFJD	Antibiotic + ST	No	10	5;6
Wei 2019 [43]	Inpatient	NR	NR	30/30	53.3/52.7	60/56.7	SFJD	Ceftriaxone (iv.gtt, 2 g/d) + ST	No	10(SFJD7)	1;4;7;9
Yao 2017 [44]	Inpatient	NR	NR	20/20	65.4/64.1	85/80	SFJD	Antibiotic + ST	No	7	1;2;7;9
Zhang 2015 [45]	Inpatient	< 48 h	NR	65/65	67/66	75.4/72.3	SFJD	Cefuroxime + ST	No	10	1;5;9
Zhang 2019 [46]	Inpatient	NR	NR	30/30	61.5/62.3	76.7/83.3	SFJD	Antibiotic + ST	No	14	1;6;7
Zhu 2018 [47]	Inpatient	< 72 h	NR	30/30	NR	63/70	SFJD	Antibiotic + ST	No	6	1;4;7;9

Notes: ^a^ The dose of SFJD was 4 capsules per time, and 3 times daily in all trials. ^b^ 1.treatment failure; 2. duration of hospital stay; 3. ICU admission; 4. time to resolution of clinical symptoms; 5. PaO2 and PaCO2; 6. lung function (FEV1/FVC ratio); 7. Infection-related index; 8. health related quality of life; 9. adverse event. *NR* not reported, *SFJD* Shufeng Jiedu capsule, *ST* symptomatic treatment, *iv.gtt* intravenous drip

### Risk of Bias assessment

Three trials reported clear randomisation concealment [40, 41, 47]. Placebo was not used in any of the studies, so there was no blinding of patients or clinicians. Loss to follow-up was rarely reported; two trials had more than 5% attrition [35, 36] (Table 2).

**Table 2:** Quality assessment: risk of bias

Study ID	Random sequence generated	Allocation concealed	Blinding		Attrition infrequent ^a^	Free of selective reporting
			Patients & clinicians	Outcome assessors		
Bian 2016 [35]	Probably yes	Probably yes	No	Probably no	Probably no	Probably yes
Hu 2018 [36]	Probably yes	Probably yes	No	Probably no	Probably no	Probably yes
Huang 2015 [37]	Probably yes	Probably yes	No	Probably no	NR	Probably yes
Li 2017a [38]	Probably yes	Probably yes	No	Probably no	NR	Probably yes
Li 2017b [39]	Probably yes	Probably yes	No	Probably no	NR	Probably yes
Tian 2018 [40]	Yes	Yes	No	Probably no	NR	No
Wang 2016 [41]	Yes	Yes	No	Probably no	NR	Probably yes
Wang 2018 [42]	Probably yes	Probably yes	No	Probably no	NR	Probably yes
Wei 2019 [43]	Probably yes	Probably yes	No	Probably no	NR	Probably yes
Yao 2017 [44]	Probably yes	Probably yes	No	Probably no	Probably yes	Probably yes
Zhang 2015 [45]	Probably yes	Probably yes	No	Probably no	NR	Probably yes
Zhang 2019 [46]	Probably yes	Probably yes	No	Probably no	NR	Probably yes
Zhu 2018 [47]	Yes	Yes	No	Probably no	NR	Probably yes

Notes: ^a^ Yes defined as less than 10% attrition to all outcome and those excluded not likely to have made a material difference in outcomes. All answers as: yes, probably yes, probably no, no. *NR* not reported

### Outcomes

No trial reported information about all-cause mortality, re-exacerbation or antibiotic dosage.

#### Treatment failure

**Fig. 2:**
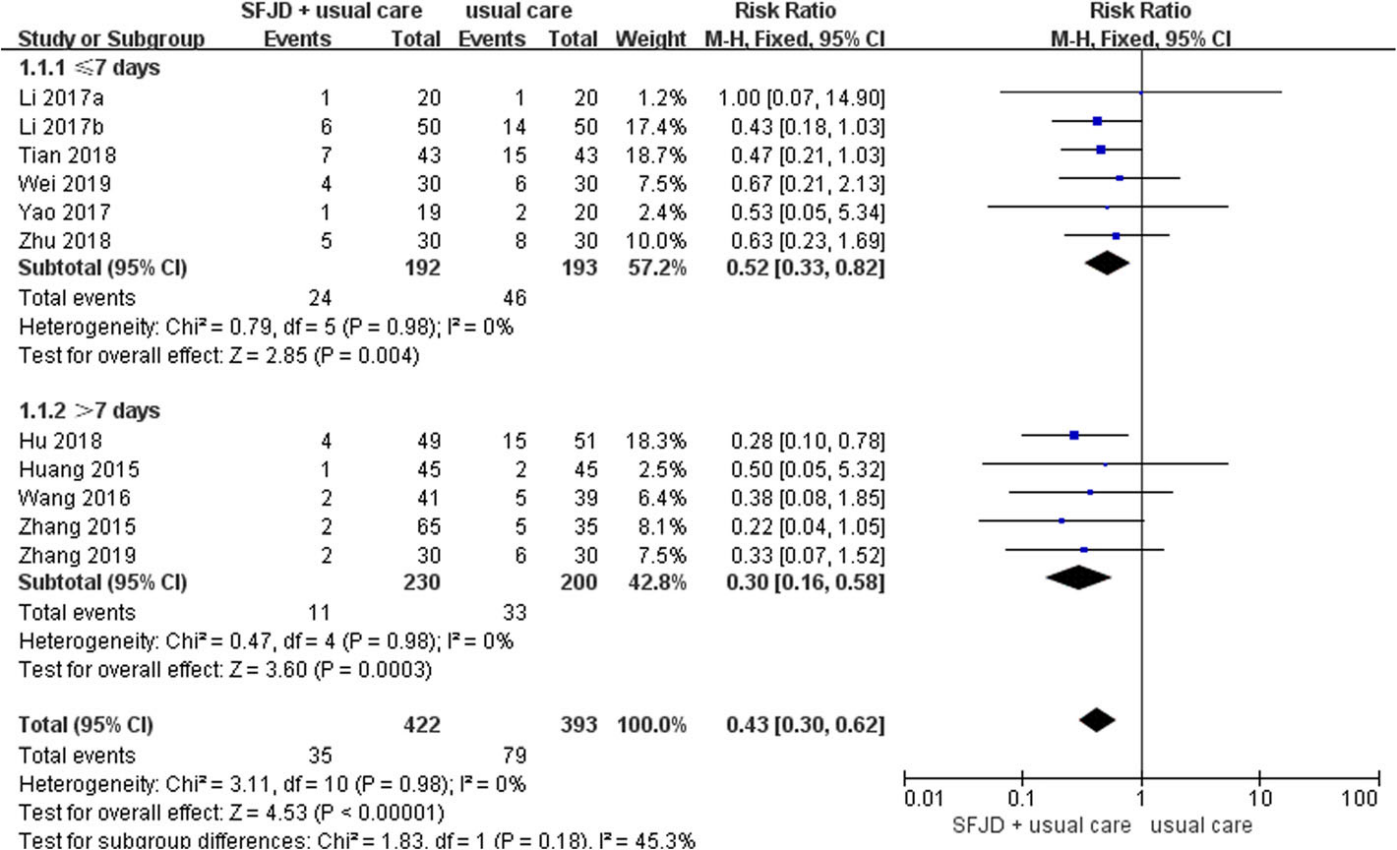
Impact of Shufeng Jiedu on treatment failure in AECOPD patients stratified by treatment duration (days). SFJD: Shufeng Jiedu capsule; AECOPD: acute exacerbations of chronic obstructive pulmonary disease

**Fig. 3:**
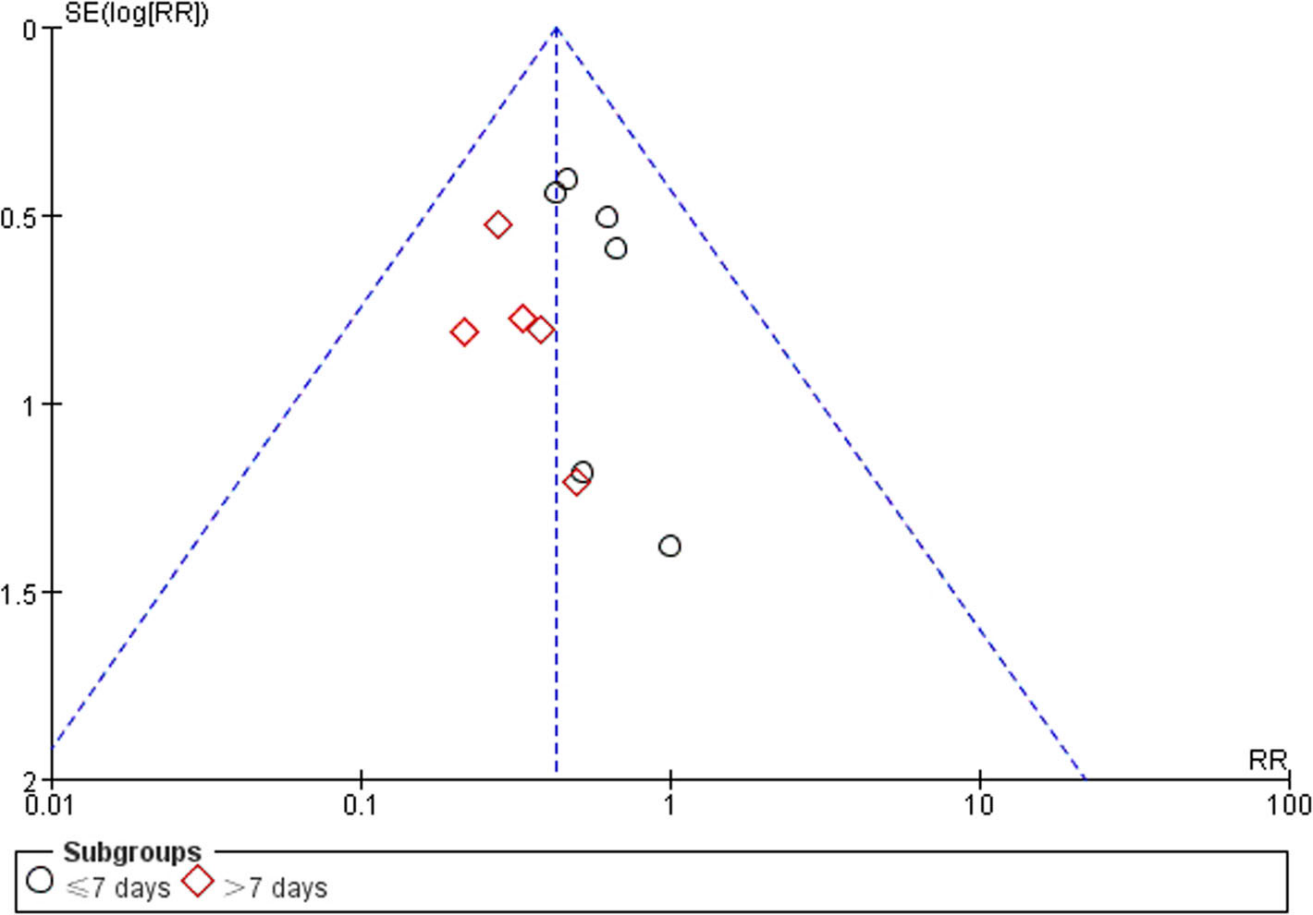
Funnel plot of treatment failure. SE: Standard error; RR: risk ratio

Eleven trials [36–41, 43–47] addressed treatment failure (details of treatment failure criteria are presented in Additional file 1: Table 2), with 35 of 422 (8.3%) patients experienced treatment failure in the SFJD group compared to 79 of 393 (20.1%) patients in the control group (RR 0.43, 95% CI 0.30 to 0.62; I^2^ = 0%; low certainty) (Fig. 2). Certainty in evidence was rated as low due to the lack of blinding and because a small number of events (Table 3). No subgroup analyses or sensitivity analyses materially changed the results (Additional file 1: Table 3, Figures 1, 2, 3, 4 and 5). The results of all subgroup analyses and sensitivity analyses were listed in the Additional file 1: Table 3. We were not able to detect publication bias for treatment failure using funnel plots (Fig. 3), Begg’s test (*P* = 0.94), and Egger’s test (*P* = 0.95).

**Table 3:** Certainty in the estimates rated according to the GRADE. Question: Shufeng Jiedu combine antibiotic and symptomatic treatment versus antibiotic combine symptomatic treatment

Certainty assessment							№ of patients		Effect		Certainty	Importance
№ of studies	Study design	Risk of bias	Inconsistency	Indirectness	Imprecision	Publication bias	SFJD + usual care	usual care	Relative (95% CI)	Absolute (95% CI)		
**Treatment failure**												
11	randomised trials	serious ^a^	not serious	not serious	serious ^b^	Undetected	35/422 (8.3%)	79/393 (20.1%)	**RR 0.43** (0.30 to 0.62)	**115 fewer per 1,000** (from 141 fewer to 76 fewer)	⨁⨁◯◯ LOW	CRITICAL
**Treatment failure - Treatment duration≤7 days**												
6	randomised trials	serious ^a^	not serious	not serious	serious ^b^	Undetected	24/192 (12.5%)	46/193 (12.8%)	**RR 0.52** (0.33 to 0.82)	**114 fewer per 1,000** (from 160 fewer to 43 fewer)	⨁⨁◯◯ LOW	CRITICAL
**Treatment failure - Treatment duration >7days**												
5	randomised trials	serious ^a^	not serious	not serious	serious ^b^	Undetected	11/230 (4.8%)	33/200 (16.5%)	**RR 0.30** (0.16 to 0.58)	**115 fewer per 1,000** (from 139 fewer to 69 fewer)	⨁⨁◯◯ LOW	CRITICAL
**Treatment failure - Mode of administration of antibiotics - iv.gtt**												
2	randomised trials	serious ^a^	not serious	not serious	serious ^b^	Undetected	10/80 (12.5%)	20/80 (25.0%)	**RR 0.50** (0.25 to 1.00)	**125 fewer per 1,000** (from 188 fewer to 0 fewer)	⨁⨁◯◯ LOW	CRITICAL
**Treatment failure - Mode of administration of antibiotics - Not reported**												
9	randomised trials	serious ^a^	not serious	not serious	serious ^b^	Undetected	25/342 (7.3%)	59/313 (18.8%)	**RR 0.41** (0.26 to 0.62)	**111 fewer per 1,000** (from 139 fewer to 72 fewer)	⨁⨁◯◯ LOW	CRITICAL
**Treatment failure - Complications - Not reported**												
11	randomised trials	serious ^a^	not serious	not serious	serious ^b^	Undetected	29/372 (7.8%)	65/343 (19.0%)	**RR 0.43** (0.29 to 0.64)	**108 fewer per 1,000** (from 135 fewer to 68 fewer)	⨁⨁◯◯ LOW	CRITICAL
**Treatment failure - Complications - AECOPD combined pulmonary infection**												
1	randomised trials	serious ^a^	not serious	not serious	serious ^b^	Undetected	6/50 (12.0%)	14/50 (28.0%)	**RR 0.43** (0.18 to 1.03)	**160 fewer per 1,000** (from 230 fewer to 8 more)	⨁⨁◯◯ LOW	CRITICAL
**Duration of hospital stay (day)**												
2	randomised trials	serious ^a^	not serious	not serious	serious ^c^	Undetected	39	40	–	MD **4.32 lower** (5.89 lower to 2.75 lower)	⨁⨁◯◯ LOW	CRITICAL

Notes: ^a^ The blinding was not used; ^b^ A small number of events; ^c^ Number of included patients is small. *SFJD* Shufeng Jiedu capsule, *AECOPD* acute exacerbations of chronic obstructive pulmonary disease, *iv.gtt* intravenous drip, *CI* Confidence interval, *RR* Risk ratio, *MD* Mean difference

#### Duration of hospital stay

Two trials [38, 44] (79 patients) demonstrated a significant reduction in the length of hospitalisation (MD − 4.32 days; − 5.89 to − 2.75 days; *I*^*2*^ = 65%; low certainty) in SFJD group (Additional file 1: Figure 6).

#### ICU admission

Only one trial [36] (120 patients) provided data on admission to an ICU for mechanical ventilation among patients not in an ICU at the time of enrolment. Six of 60 patients in the SFJD group and seven of 60 patients in the control group were admitted to an ICU respectively.

#### Time to resolution of clinical symptoms

Two trials [43, 47] (79 patients) evaluated time to resolution of fever. The pooled results showed no significant difference in the length of fever (SMD -1.46, − 3.24 to 0.32; *I*^*2*^ = 90%; very low certainty) between SFJD group and control group (Additional file 1: Table 4). There was a shorter time to resolution of sputum (two trials, 160 patients, MD − 1.68 days; − 2.21 to − 1.16 days; *I*^*2*^ = 0%; low certainty) and crackles (two trials, 160 patients, MD − 1.23 days; − 2.12 to − 0.34 days; *I*^*2*^ = 70%; very low certainty) in the SFJD group [39, 43] (Additional file 1: Table 4). In one trial [39] (100 patients), SFJD group showed significantly shorter time to resolution of cough (MD − 1.20 days, − 1.69 to − 0.71 days).

#### PaO2 and PaCO2

Significant benefits were found for improvement in PaO2 (4 trials, 390 patients, MD 7.69 mmHg, 3.68 to 11.70 mmHg; I^2^ = 92%; very low certainty) and reduction in PaCO2 at end of therapy (4 trials, 390 patients, MD − 3.73 mmHg, − 6.01 to − 1.45 mmHg; *I*^*2*^ = 69%; very low certainty) in SFJD group compared to control group (Additional file 1: Table 4).

#### FEV1/FVC ratio

Four trials [35, 36, 42, 46] (307 patients) examined the FEV1/FVC ratio. The pooled results demonstrated a significant increase in the FEV1/FVC ratio (MD 4.83, 2.56 to 7.10; *I*^*2*^ = 57%; very low certainty) in the SFJD group. Subgroup analysis did not have a meaningful impact on the results.

#### White cell counts and inflammatory markers

Three trials [43, 44, 47] (159 patients) investigated white blood cell counts in patients at the end of treatment. Pooled results showed that SFJD group had lower white blood cell count (MD -1.78 × 10^9^/L, − 3.16 to − 0.40 × 10^9^/L; *I*^*2*^ = 79%; very low certainty) (Additional file 1: Table 4).

Two trials [44, 47] (99 patients) provided information on the proportion of neutrophils at the end of treatment. Meta-analysis showed that patients with AECOPD treated with SFJD had lower proportion of neutrophils (MD -3.69%, − 4.65 to − 2.73%; *I*^*2*^ = 0%; low certainty) (Additional file 1: Table 4).

Six trials [35, 37, 39, 43, 44, 46, 47] reported on C-reactive protein (CRP). Patients treated with SFJD combined with usual care had lower serum CRP levels at end of therapy compared to usual care patients (426 patients, MD − 5.29 mg/ L, − 8.45 to − 2.14 mg/ L; *I*^*2*^ = 98%; very low certainty) (Additional file 1: Table 4).

Two trials [39, 43] (160 patients) provided data on procalcitonin among patients. Pooled results showed no significantly difference (MD − 0.61 ng/L, − 1.26 to 0.04 ng/L; *I*^*2*^ = 93%; very low certainty) at the end of treatment in the SFJD group and the usual care group (Additional file 1: Table 4).

#### Health related quality of life

Only one trial [35] (77 patients) investigated quality of life. COPD Assessment Test (CAT) score reduced from 21.7 to 15.3 in patients treated with SFJD and from 22.3 to 17.3 in the control group (MD between two groups at the end of treatment − 2.00, − 4.30 to − 0.30). Previous study has shown that the minimum clinical important difference is a decrease in CAT score of 2 points [48].

#### Adverse events

No difference in the incidence of adverse events was found between those who did and did not take SFJD (8 trials; 605 patients; RR 1.41, 0.46 to 4.33; *I*^*2*^ = 0%; low certainty). Subgroup analyses did not have a meaningful impact on the results (Additional file 1: Table 4). The adverse events reported in these trials included transient gastrointestinal tract reactions, including nausea and diarrhoea. No serious adverse event was reported.

## Discussion

### Main findings

Our systematic review found low certainty evidence suggesting possible benefits from SFJD in combination with usual care for patients with AECOPD, compared to usual care. However, these effects can only be reported with low or very low certainty due to high risk of bias, mainly from lack of blinding, and the insufficient number of trials. Our findings suggested a significant reduction in the rate of treatment failure (57% reduction in the risk of treatment failure). We found an estimated reduction of 4 days in hospital stay from SFJD with usual care. We also found low certainty evidence that SFJD as adjuvant therapy may offer benefits in physiological outcomes: PaO2, PaCO2, FEV1/FVC ratio, white cell counts and inflammatory markers (low to very low certainty). We found no evidence of serious adverse events associated with use of SFJD.

### Strengths and limitations

The review was registered in advance and followed rigorous methodology with pre-specified eligibility criteria, a comprehensive search with English and Chinese databases included without language restrictions, and assessment of eligibility and risk of bias in duplicate. We conducted some pre-defined plausible subgroup and sensitivity analyses and applied GRADE criteria to determine certainty of evidence.

Twelve of the 13 trials we included focused on inpatients, and all trials combined SFJD with antibiotics and symptomatic treatment, which is in line with routine clinical practice. In addition, the daily dosing regimen of SFJD was the same in all trials.

There are several limitations: No trials included placebo. Usual care was not standardised across trials, the characteristics of patients varied and phenotyping was poor so it was difficult to identify a responder population. All trials had high risk of bias in the blinding domain, and most trials had unclear risk of bias in attrition. Additionally, inferences were also limited for some outcomes with a small number of patients and events. Most GRADE evidence certainty was low or very low which suggests that readers should be legitimately concerned with the evidence. However, our findings were robust to a variety of subgroup and sensitivity analyses.

### Relation to prior research work

We identified one published systematic review [49] (in Chinese) of SFJD for AECOPD. This previous systematic review did not register their protocol, and GRADE was not utilised to evaluate the confidence of evidence. The nine trials identified in this previous systematic review were all included in our review. Our results are consistent with the prior systematic review. Inclusion of recent trials allowed us to address additional outcomes, including ICU admission, health related quality of life and time to resolution of clinical symptoms, as well as to provide assessment of included evidence certainty.

### Implications

Our results support the need for a future blinded RCT of SFJD for AECOPD. All trials had a high risk of bias; and as very few trials included outpatients it is unclear whether our findings can be generalised to this setting. Similar effects across outcomes (treatment failure, length of hospital stay, PaO2and PaCO2, FEV1/FVC ratio, clinical symptoms and infection-related index) strengthened the credibility of the findings.

One trial [39] required that participants had to have pulmonary infection confirmed by guidelines for diagnosis and treatment of community-acquired pneumonia [50]. Although subgroup analyses showed that results from this trial were not significantly different from other trials, it remains possible that the effects of SFJD vary by underlying pulmonary infection.

The apparent benefits of SFJD combined with antibiotic and symptomatic treatment in AECOPD are considered important – an absolute reduction in risk of treatment failure (11.8% lower) and shortening of hospital stay (approximately 4 days fewer) without identifiable long-term or serious adverse events compared to antibiotic and symptomatic treatment.

## Conclusion

Published trials suggested positive effects of SFJD in combination of antibiotics and symptomatic treatments on reducing treatment failure, duration of hospitalisation, symptoms. However, due to the high risk of bias and the limited number of trials, the findings were inconclusive. Further high-quality, rigorously conducted double-blind, placebo-controlled trials are warranted.

## Supplementary information

**Additional file 1: Table 1.** Herbal compositions of Shufeng Jiedu capsule. **Table 2.** Definitions of treatment failure in 11 trials. **Table 3.** Subgroup analyses and sensitivity analyses for treatment failure with Shufeng Jiedu for AECOPD patients. **Figure 1.** Impact of Shufeng Jiedu capsule on treatment failure in AECOPD patients stratified by mode of administration of antibiotics. **Figure 2.** Impact of Shufeng Jiedu on treatment failure in AECOPD patients stratified by complications. **Figure 3.** Impact of Shufeng Jiedu on treatment failure in AECOPD patients stratified by clear or unclear randomization concealment. **Figure 4.** Impact of Shufeng Jiedu on treatment failure in AECOPD patients stratified by reported loss to follow up or not reported. **Figure 5.** Impact of Shufeng Jiedu on treatment failure in AECOPD patients used worst plausible assumptions of patients lost to follow up or not used. **Figure 6.** Impact of Shufeng Jiedu on duration of hospital stay (days) in AECOPD patients. **Table 4.** Certainty in the estimates rated according to the GRADE.

## Data Availability

All data used in this review are included in this published article.

## Abbreviations

AECOPD: Acute exacerbation of chronic obstructive pulmonary disease
CAT: COPD Assessment Test
CI: Confidence intervals
CNKI: China National Knowledge Infrastructure
COPD: Chronic obstructive pulmonary disease
FEV1: Forced expiratory volume in 1 s
FVC: Forced vital capacity
GRADE: Grading of Recommendations Assessment, Development and Evaluation
ICU: Intensive care unit
MD: Mean difference
PaCO2: Partial pressure of carbon dioxide
PaO2: Partial pressure of oxygen
PRISMA: Preferred Reporting Items for Systematic Reviews and Meta-Analysis
RCTs: Randomised controlled trials
RR: Risk ratio
SFJD: Shufeng Jiedu
SMD: Standard mean difference
VIP: Chinese Scientific Journal Database
