# Supplementary material for: Shufeng Jiedu capsules for treating acute exacerbations of chronic obstructive pulmonary disease: a systematic review and meta-analysis
Source: BMC Complement Med Ther. 2020 May 24;20:151. doi: 10.1186/s12906-020-02924-5 (PMC7245765; doi:10.1186/s12906-020-02924-5)
Supplement: Supplementary file 1 — Additional file 1: Table 1. Herbal compositions of Shufeng Jiedu capsule. Table 2. Definitions of treatment failure in 11 trials. Table 3. Subgroup analyses and sensitivity analyses for treatment failure with Shufeng Jiedu for AECOPD patients. Figure 1. Impact of Shufeng Jiedu capsule on treatment failure in AECOPD patients stratified by mode of administration of antibiotics. Figure 2. Impact of Shufeng Jiedu on treatment failure in AECOPD patients stratified by complications. Figure 3. Impact of Shufeng Jiedu on treatment failure in AECOPD patients stratified by clear or unclear randomization concealment. Figure 4. Impact of Shufeng Jiedu on treatment failure in AECOPD patients stratified by reported loss to follow up or not reported. Figure 5. Impact of Shufeng Jiedu on treatment failure in AECOPD patients used worst plausible assumptions of patients lost to follow up or not used. Figure 6. Impact of Shufeng Jiedu on duration of hospital stay (days) in AECOPD patients. Table 4. Certainty in the estimates rated according to the GRADE. [file 12906_2020_2924_MOESM1_ESM.docx]

**Additional file 1**

**Table 1.** Herbal compositions of Shufeng Jiedu capsule

| Latin botanic Name | Chinese Pinyin Name |
| --- | --- |
| *Fallopia japonica* (Houtt.) | Hu Zhang |
| *Forsythia suspense* (Thunb.) | Lian Qiao |
| *Isatis indigotica* (Fort.) | Ban Lan Gen |
| *Bupleurum chinense* (DC.) | Chai Hu |
| *Patrinia scabiosaefolia*(Fisch.) | Bai Jiang Cao |
| *Verbena officinialis* (L.) | Ma Bian Cao |
| *Phragmites communis* (Trin.) | Lu Gen |
| *Glycyrrhiza uralensis* (Fisch.) | Gan Cao |

**Table 2.** Definitions of treatment failure in 11 trials

| Study ID | Criteria of treatment failure |
| --- | --- |
| Hu 2018 ^[36]^ | No resolution of symptoms and signs |
| Huang 2015 ^[37]^ | No resolution of symptoms and signs |
| Li 2017a ^[38]^ | No resolution of symptoms and signs |
| Li 2017b ^[39]^ | No resolution or deterioration of symptoms and signs |
| Tian 2018 ^[40]^ | No resolution or deterioration of symptoms and signs |
| Wang 2016 ^[41]^ | No resolution of symptoms and signs |
| Wei 2019 ^[43]^ | No resolution of symptoms and signs |
| Yao 2017 ^[44]^ | Nothing changed obviously after treatment |
| Zhang 2015 ^[45]^ | No resolution or deterioration of symptoms and signs |
| Zhang 2019 ^[46]^ | No resolution of symptoms and signs |
| Zhu 2018 ^[47]^ | No resolution or deterioration of symptoms and signs |

**Table 3.** Subgroup analyses and sensitivity analyses for treatment failure with Shufeng Jiedu for AECOPD patients

| Analytic factors | Subgroup/sensitivity analyses | n studies | RR (95% CI) | *P­­-*value |
| --- | --- | --- | --- | --- |
| Severity | Inpatients | 11 |  |  |
|  | Outpatients | 0 |  |  |
| Treatment duration | ≤7days | 6 | 0.52 (0.33, 0.82) |  |
|  | >7 days | 5 | 0.30 (0.16, 0.58) | 0.18 |
| Mode of administration of antibiotics | iv.gtt | 2 | 0.50 (0.25, 1.00) |  |
|  | Not reported | 9 | 0.41 (0.26, 0.62) | 0.62 |
| Complications | Not reported | 11 | 0.43 (0.29, 0.64) |  |
|  | AECOPD combined pulmonary infection | 1 | 0.43 (0.18, 1.03) | 1 |
| Number of centres | Multi-centre | 0 |  |  |
|  | Single centre | 11 |  |  |
| Randomization concealment | Clear | 3 | 0.50 (0.28, 0.88) |  |
|  | Unclear | 8 | 0.39 (0.24, 0.63) | 0.54 |
| Placebo | Used | 0 |  |  |
|  | Not used | 11 |  |  |
| Loss-to-follow-up | Reported | 2 | 0.31 (0.12, 0.78) |  |
|  | Not reported | 9 | 0.46 (0.31, 0.69) | 0.43 |
| Assumed worst plausible case results | Used | 11 | 0.52 (0.38, 0.71) |  |
|  | Not used | 11 | 0.43 (0.30, 0.62) | 0.45 |

Notes: AECOPD: acute exacerbations of chronic obstructive pulmonary disease; RR: risk ratio; CI: confidence interval; iv.gtt: Intravenous drip.

**
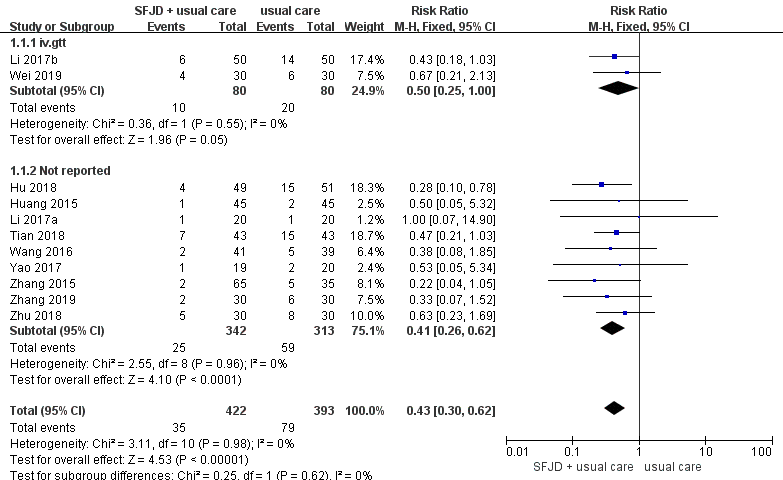
**

**Figure 1.** Impact of Shufeng Jiedu capsule on treatment failure in AECOPD patients stratified by mode of administration of antibiotics

Notes: SFJD: Shufeng Jiedu capsule; AECOPD: acute exacerbations of chronic obstructive pulmonary disease; iv.gtt: intravenous drip.

**
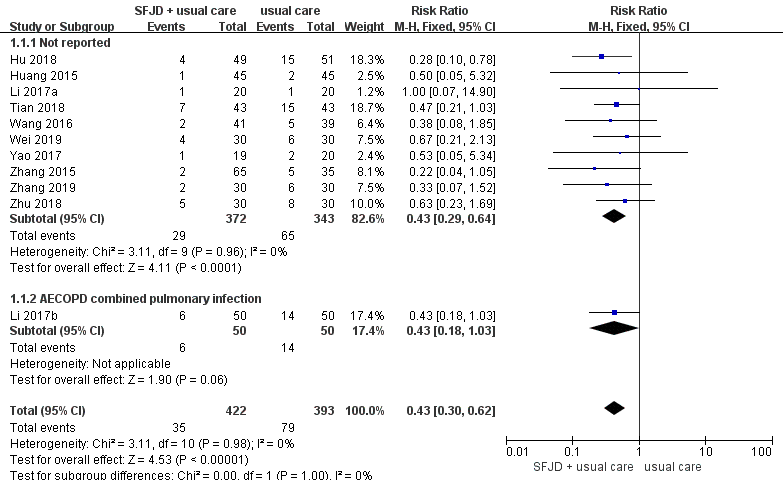
**

**Figure 2.** Impact of Shufeng Jiedu on treatment failure in AECOPD patients stratified by complications

Notes: SFJD: Shufeng Jiedu capsule; AECOPD: acute exacerbations of chronic obstructive pulmonary disease.

**
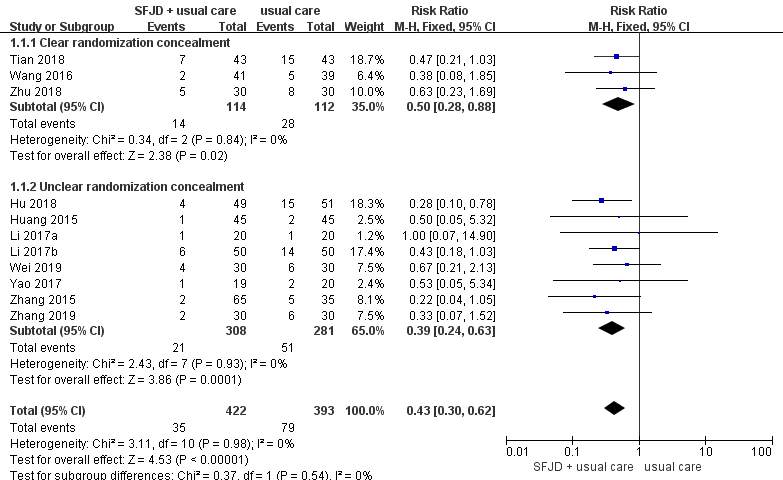
**

**Figure 3.** Impact of Shufeng Jiedu on treatment failure in AECOPD patients stratified by clear or unclear randomization concealment

Notes: SFJD: Shufeng Jiedu capsule; AECOPD: acute exacerbations of chronic obstructive pulmonary disease.

**
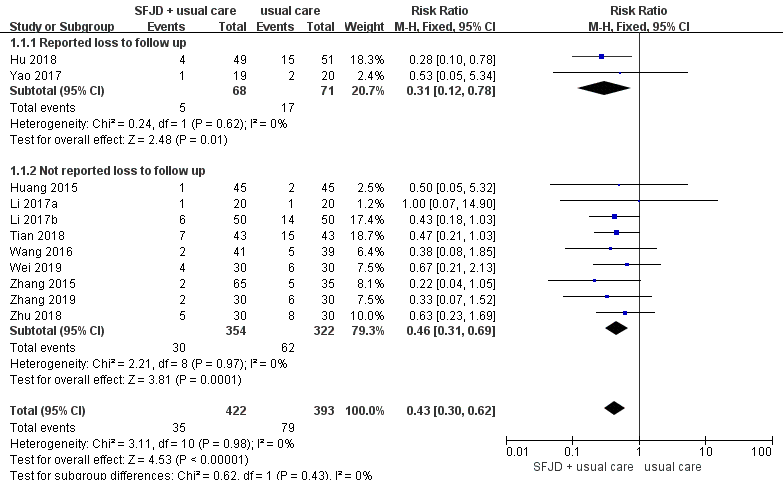
**

**Figure 4.** Impact of Shufeng Jiedu on treatment failure in AECOPD patients stratified by reported loss to follow up or not reported

Notes: SFJD: Shufeng Jiedu capsule; AECOPD: acute exacerbations of chronic obstructive pulmonary disease.

**
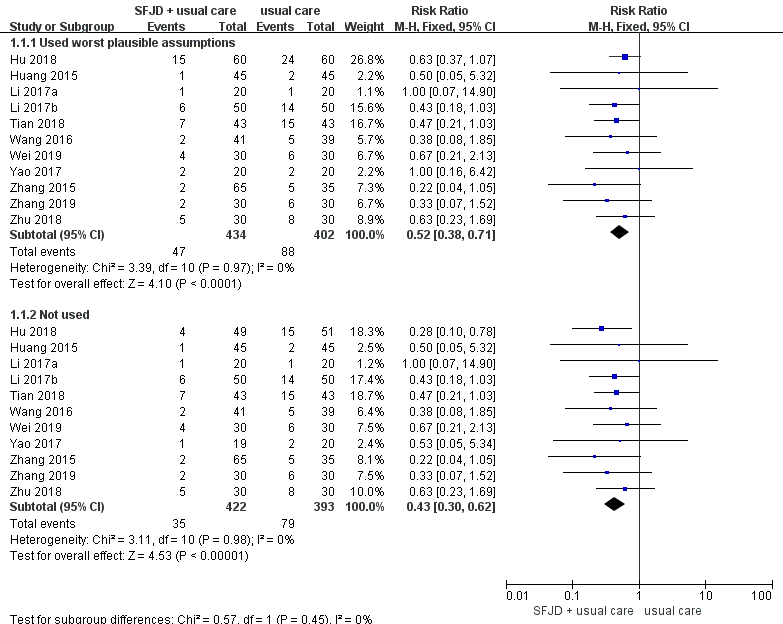
**

**Figure 5.** Impact of Shufeng Jiedu on treatment failure in AECOPD patients used worst plausible assumptions of patients lost to follow up or not used

Notes: SFJD: Shufeng Jiedu capsule; AECOPD: acute exacerbations of chronic obstructive pulmonary disease.

**
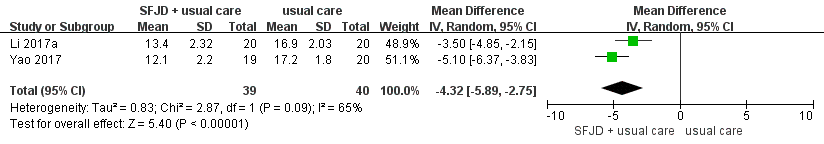
**

**Figure 6.** Impact of Shufeng Jiedu on duration of hospital stay (days) in AECOPD patients

Notes: SFJD: Shufeng Jiedu capsule; AECOPD: acute exacerbations of chronic obstructive pulmonary disease.

**Table 4.** Certainty in the estimates rated according to the GRADE

Question: Shufeng Jiedu combined with antibiotic and symptomatic treatment versus antibiotic combined with symptomatic treatment

| **Certainty assessment** | | | | | | | **№ of patients** | | **Effect** | | **Certainty** | **Importance** |
| --- | --- | --- | --- | --- | --- | --- | --- | --- | --- | --- | --- | --- |
| **№ of studies** | **Study design** | **Risk of bias** | **Inconsistency** | **Indirectness** | **Imprecision** | **Publication bias** | **SFJD + usual care** | **usual care** | **Relative (95% CI)** | **Absolute (95% CI)** |  |  |
| **ICU admission** | | | | | | | | | | | | |
| 1 | randomised trials | serious ^a^ | not serious | not serious | serious ^d^ | Undetected | 6/60 (10.0%) | 7/60 (11.7%) | **RR 0.84** (0.27 to 2.67) | **19 fewer per 1,000**  (from 85 fewer to 195 more) | ⨁⨁◯◯ LOW | IMPORTANT |
| **Time to resolution of fever** | | | | | | | | | | | | |
| 2 | randomised trials | serious ^a^ | serious ^b^ | not serious | serious ^c^ | Undetected | 40 | 39 | - | SMD **1.46 lower**  (3.24 lower to 0.32 higher) | ⨁◯◯◯ VERY LOW | IMPORTANT |
| **Time to resolution of sputum (day)** | | | | | | | | | | | | |
| 2 | randomised trials | serious ^a^ | not serious | not serious | serious ^c^ | Undetected | 80 | 80 | - | MD **1.68 lower** (2.21 lower to 1.16 lower) | ⨁⨁◯◯ LOW | IMPORTANT |
| **Time to resolution of** **crackles (day)** | | | | | | | | | | | | |
| 2 | randomised trials | serious ^a^ | serious ^b^ | not serious | serious ^c^ | Undetected | 80 | 80 | - | MD **1.23 lower**  (2.12 lower to 0.34 lower) | ⨁◯◯◯ VERY LOW | IMPORTANT |
| **Partial pressure of oxygen dissolved in arterial blood (PaO2) (mmHg)** | | | | | | | | | | | | |
| 4 | randomised trials | serious ^a^ | serious ^b^ | not serious | serious ^c^ | Undetected | 194 | 196 | - | MD **7.69 higher** (3.68 higher to 11.70 higher) | ⨁◯◯◯ VERY LOW | IMPORTANT |
| **Partial pressure of carbon dioxide dissolved in arterial blood (PaCO2) (mmHg)** | | | | | | | | | | | | |
| 4 | randomised trials | serious ^a^ | serious ^b^ | not serious | serious ^c^ | Undetected | 194 | 196 | - | MD **3.73 lower** (6.01 lower to 1.45 lower) | ⨁◯◯◯ VERY LOW | IMPORTANT |
| **FEV1/FVC ratio (%)** | | | | | | | | | | | | |
| 4 | randomised trials | serious ^a^ | serious ^b^ | not serious | serious ^c^ | Undetected | 157 | 150 | - | MD **4.83 higher** (2.56 higher to 7.1 higher) | ⨁◯◯◯ VERY LOW | IMPORTANT |
| **FEV1/FVC ratio (%) – Severity - Inpatients** | | | | | | | | | | | | |
| 3 | randomised trials | serious ^a^ | serious ^b^ | not serious | serious ^c^ | Undetected | 114 | 116 | - | MD **5.56 higher** (2.8 higher to 8.32 higher) | ⨁◯◯◯ VERY LOW | IMPORTANT |
| **FEV1/FVC ratio (%) – Severity - Outpatients** | | | | | | | | | | | | |
| 1 | randomised trials | serious ^a^ | not serious | not serious | serious ^c^ | Undetected | 43 | 34 | - | MD **2.58 higher** (0.88 lower to 6.04 higher) | ⨁⨁◯◯ LOW | IMPORTANT |
| **FEV1/FVC ratio (%) - Treatment duration - ≤7days** | | | | | | | | | | | | |
| 1 | randomised trials | serious ^a^ | not serious | not serious | serious ^c^ | Undetected | 43 | 34 | - | MD **2.58 higher** (0.88 lower to 6.04 higher) | ⨁⨁◯◯ LOW | IMPORTANT |
| **FEV1/FVC ratio (%) - Treatment duration - >7 days** | | | | | | | | | | | | |
| 3 | randomised trials | serious ^a^ | serious ^b^ | not serious | serious ^c^ | Undetected | 114 | 116 | - | MD **5.56 higher** (2.8 higher to 8.32 higher) | ⨁◯◯◯ VERY LOW | IMPORTANT |
| **White blood cell count (×10^9/L)** | | | | | | | | | | | | |
| 3 | randomised trials | serious ^a^ | serious ^b^ | not serious | serious ^c^ | Undetected | 79 | 80 | - | MD **1.78 lower** (3.16 lower to 0.4 lower) | ⨁◯◯◯ VERY LOW | IMPORTANT |
| **White blood cell count (×10^9/L) - Treatment duration - ≤7days** | | | | | | | | | | | | |
| 2 | randomised trials | serious ^a^ | serious ^b^ | not serious | serious ^c^ | Undetected | 49 | 50 | - | MD **1.53 lower** (4.18 lower to 1.12 higher) | ⨁◯◯◯ VERY LOW | IMPORTANT |
| **White blood cell count (×10^9/L) - Treatment duration - >7 days** | | | | | | | | | | | | |
| 1 | randomised trials | serious ^a^ | not serious | not serious | serious ^c^ | Undetected | 30 | 30 | - | MD **2.01 lower** (3.08 lower to 0.94 lower) | ⨁⨁◯◯ LOW | IMPORTANT |
| **White blood cell count (×10^9/L) - Mode of administration of antibiotics - iv.gtt** | | | | | | | | | | | | |
| 1 | randomised trials | serious ^a^ | not serious | not serious | serious ^c^ | Undetected | 30 | 30 | - | MD **2.01 lower** (3.08 lower to 0.94 lower) | ⨁⨁◯◯ LOW | IMPORTANT |
| **White blood cell count (×10^9/L) - Mode of administration of antibiotics - Not reported** | | | | | | | | | | | | |
| 2 | randomised trials | serious ^a^ | serious ^b^ | not serious | serious ^c^ | Undetected | 49 | 50 | - | MD **1.53 lower** (4.18 lower to 1.12 higher) | ⨁◯◯◯ VERY LOW | IMPORTANT |
| **Proportion of neutrophilic granulocytes (%)** | | | | | | | | | | | | |
| 2 | randomised trials | serious ^a^ | not serious | not serious | serious ^c^ | Undetected | 49 | 50 | - | MD **3.69 lower** (4.65 lower to 2.73 lower) | ⨁⨁◯◯ LOW | IMPORTANT |
| **C-reactive protein (CRP) (mg/L)** | | | | | | | | | | | | |
| 6 | randomised trials | serious ^a^ | serious ^b^ | not serious | serious ^c^ | Undetected | 217 | 209 | - | MD **5.29 lower** (8.45 lower to 2.14 lower) | ⨁◯◯◯  VERY LOW | IMPORTANT |
| **C-reactive protein (CRP) (mg/L) – Severity - Inpatients** | | | | | | | | | | | | |
| 5 | randomised trials | serious ^a^ | serious ^b^ | not serious | serious ^c^ | Undetected | 174 | 175 | - | MD **5.63 lower** (9.11 lower to 2.15 lower) | ⨁◯◯◯ VERY LOW | IMPORTANT |
| **C-reactive protein (CRP) (mg/L) – Severity - Outpatients** | | | | | | | | | | | | |
| 1 | randomised trials | serious ^a^ | not serious | not serious | serious ^c^ | Undetected | 43 | 34 | - | MD **3.41 lower** (6.26 lower to 0.56 lower) | ⨁⨁◯◯ LOW | IMPORTANT |
| **C-reactive protein (CRP) (mg/L) - Treatment duration - ≤7days** | | | | | | | | | | | | |
| 3 | randomised trials | serious ^a^ | serious ^b^ | not serious | serious ^c^ | Undetected | 92 | 84 | - | MD **7.15 lower** (11.41 lower to 2.90 lower) | ⨁◯◯◯ VERY LOW | IMPORTANT |
| **C-reactive protein (CRP) (mg/L) - Treatment duration - >7 days** | | | | | | | | | | | | |
| 3 | randomised trials | serious ^a^ | serious ^b^ | not serious | serious ^c^ | Undetected | 125 | 125 | - | MD **3.52 lower** (7.83 lower to 0.78 lower) | ⨁◯◯◯ VERY LOW | IMPORTANT |
| **C-reactive protein (CRP) (mg/L) - Mode of administration of antibiotics - iv.gtt** | | | | | | | | | | | | |
| 2 | randomised trials | serious ^a^ | serious ^b^ | not serious | serious ^c^ | Undetected | 80 | 80 | - | MD **9.34 lower** (13.19 lower to 5.48 lower) | ⨁◯◯◯ VERY LOW | IMPORTANT |
| **C-reactive protein (CRP) (mg/L) - Mode of administration of antibiotics - Not reported** | | | | | | | | | | | | |
| 4 | randomised trials | serious ^a^ | serious ^b^ | not serious | serious ^c^ | Undetected | 137 | 129 | - | MD **3.19 lower** (5.83 lower to 0.55 lower) | ⨁◯◯◯ VERY LOW | IMPORTANT |
| **C-reactive protein (CRP) (mg/L) - Complications - Not reported** | | | | | | | | | | | | |
| 5 | randomised trials | serious ^a^ | serious ^b^ | not serious | serious ^c^ | Undetected | 167 | 159 | - | MD **4.85 lower** (8.85 lower to 0.86 lower) | ⨁◯◯◯ VERY LOW | IMPORTANT |
| **C-reactive protein (CRP) (mg/L) - Complications - AECOPD combined pulmonary infection** | | | | | | | | | | | | |
| 1 | randomised trials | serious ^a^ | not serious | not serious | serious ^c^ | Undetected | 50 | 50 | - | MD **7.4 lower** (8.15 lower to 6.65 lower) | ⨁⨁◯◯ LOW | IMPORTANT |
| **Procalcitonin (ng/L)** | | | | | | | | | | | | |
| 2 | randomised trials | serious ^a^ | serious ^b^ | not serious | serious ^c^ | Undetected | 80 | 80 | - | MD **0.61 lower**  (1.26 lower to 0.04 higher) | ⨁◯◯◯ VERY LOW | IMPORTANT |
| **COPD Assessment Test (CAT) score** | | | | | | | | | | | | |
| 1 | randomised trials | serious ^a^ | not serious | not serious | serious ^c^ | Undetected | 43 | 34 | - | MD **2.00 lower** (4.30 lower to 0.30 lower) | ⨁⨁◯◯ LOW | IMPORTANT |
| **Adverse events** | | | | | | | | | | | | |
| 8 | randomised trials | serious ^a^ | not serious | not serious | serious ^d^ | Undetected | 6/302 (2.0%) | 4/303 (1.3%) | **RR 1.41** (0.46 to 4.33) | **5 more per 1,000**  (from 7 fewer to 42 more) | ⨁⨁◯◯ LOW | CRITICAL |
| **Adverse events - Treatment duration - ≤7days** | | | | | | | | | | | | |
| 4 | randomised trials | serious ^a^ | not serious | not serious | serious ^d^ | Undetected | 4/122 (3.3%) | 2/123 (1.6%) | **RR 1.82** (0.41 to 8.21) | **13 more per 1,000**  (from 10 fewer to 117 more) | ⨁⨁◯◯ LOW | CRITICAL |
| **Adverse events - Treatment duration - >7 days** | | | | | | | | | | | | |
| 4 | randomised trials | serious ^a^ | not serious | not serious | serious ^d^ | Undetected | 2/180 (1.1%) | 2/180 (1.1%) | **RR 1.00** (0.18 to 5.64) | **0 fewer per 1,000**  (from 9 fewer to 52 more) | ⨁⨁◯◯ LOW | CRITICAL |
| **Adverse events - Mode of administration of antibiotics - iv.gtt** | | | | | | | | | | | | |
| 2 | randomised trials | serious ^a^ | not serious | not serious | serious ^d^ | Undetected | 0/80 (0.0%) | 0/80 (0.0%) | Not estimable |  | ⨁⨁◯◯ LOW | CRITICAL |
| **Adverse events - Mode of administration of antibiotics - Not reported** | | | | | | | | | | | | |
| 6 | randomised trials | serious ^a^ | not serious | not serious | serious ^d^ | Undetected | 6/222 (2.7%) | 4/223 (1.8%) | **RR 1.41** (0.46 to 4.33) | **6 more per 1,000**  (from 8 fewer to 49 more) | ⨁⨁◯◯ LOW | CRITICAL |
| **Adverse events- Complications - Not reported** | | | | | | | | | | | | |
| 7 | randomised trials | serious ^a^ | not serious | not serious | serious ^d^ | Undetected | 6/252 (2.4%) | 4/253 (1.6%) | **RR 1.41** (0.46 to 4.33) | **6 more per 1,000**  (from 9 fewer to 53 more) | ⨁⨁◯◯ LOW | CRITICAL |
| **Adverse events - Complications - AECOPD combined pulmonary infection** | | | | | | | | | | | | |
| 1 | randomised trials | serious ^a^ | not serious | not serious | serious ^d^ | Undetected | 0/50 (0.0%) | 0/50 (0.0%) | Not estimable |  | ⨁⨁◯◯ LOW | CRITICAL |

Notes: a. The blinding was not used; b. I square value is large; c. Number of patients included is small; d. A small number of events. SFJD: Shufeng Jiedu capsule; AECOPD: acute exacerbations of chronic obstructive pulmonary disease; iv.gtt: intravenous drip; CI: Confidence interval; RR: Risk ratio; MD: Mean difference; SMD: Standardised mean difference.
